# Midlife loneliness profile predicting old‐age cognition, and moderating effects of personality

**DOI:** 10.1002/alz70861_108750

**Published:** 2025-12-23

**Authors:** Anni Varjonen, Toni T Saari, Sari Aaltonen, Teemu Palviainen, Paula Iso‐Markku, Juha O. Rinne, Jaakko Kaprio, Eero Vuoksimaa

**Affiliations:** ^1^ University of Helsinki, Helsinki Finland; ^2^ Helsinki University Hospital, Helsinki Finland; ^3^ Turku University Hospital, Turku Finland

## Abstract

**Background:**

Loneliness—a mismatch between desired and existing social relationships—has been linked to an increased dementia risk, but the nature of this association remains unclear and may involve reverse causation. Personality traits, such as higher neuroticism and lower extraversion, are also associated with both dementia risk and loneliness. Few studies, however, have accounted for personality when examining the relationship between loneliness and cognitive aging. This study aims to clarify the association between midlife loneliness and late‐life cognition by considering the potential moderating role of personality, using a long follow‐up up to 23 years.

**Method:**

Participants from the older Finnish Twin Cohort completed questionnaires in 1975, 1981, and 1990. Current feeling of loneliness was measured in all questionnaires with one item with three response options (very lonely, fairly lonely, not lonely). Neuroticism and extraversion were based on an abbreviated Eysenck’s Personality Inventory in 1975 and 1981. Covariates included sex, age, education, Cardiovascular Risk Factors, Aging and Dementia score, alcohol use, smoking status, depression status, social contact frequency, and marital status. Cognitive data were collected in 2013–2017 from 1814 individuals aged 71–79 (mean age 34 in 1975) using the Telephone Interview for Cognitive Status–modified (TICS‐m). ApoE status was determined from DNA arrays.

**Result:**

Participants were grouped as consistently lonely (*n* =202), never lonely (*n* =1238), or with varying loneliness (*n* =127). Loneliness profile and extraversion did not predict cognition, after adjustment for covariates. Higher neuroticism predicted worse cognition when adjusting for age, sex, and education (β = ‐0.127, *p* = 0.045), one‐point increase in neuroticism was linked to a 0.13‐point decrease in TICS‐m (0–50), but this was not significant after adjusting for other covariates. No significant interaction effects were found between loneliness profile, personality traits, and cognition. Covariates age, education, depression status, social contact frequency, and ApoE status consistently predicted cognitive scores.

**Conclusion:**

Midlife loneliness was not associated with late‐life cognitive functioning. While neuroticism showed a modest link to lower cognition, this did not persist after including all covariates. These findings suggest that loneliness and personality in midlife may not be independent risk factors for late‐life cognitive decline when broader health and lifestyle factors are considered.

